# Chondrocytes from Osteoarthritis Patients Adopt Distinct Phenotypes in Response to Central T_H_1/T_H_2/T_H_17 Cytokines

**DOI:** 10.3390/ijms22179463

**Published:** 2021-08-31

**Authors:** Antti Pemmari, Tiina Leppänen, Mari Hämäläinen, Teemu Moilanen, Eeva Moilanen

**Affiliations:** 1 The Immunopharmacology Research Group, Faculty of Medicine and Health Technology, University of Tampere and Tampere University Hospital, 33100 Tampere, Finland; antti.pemmari@tuni.fi (A.P.); tiina.leppanen@tuni.fi (T.L.); mari.hamalainen@tuni.fi (M.H.); 2 Coxa Hospital for Joint Replacement, 33520 Tampere, Finland; teemu.moilanen@coxa.fi

**Keywords:** chondrocyte, IL-1β, IFNγ, IL-17, IL-4, RNA-Seq

## Abstract

Chronic low-grade inflammation plays a central role in the pathogenesis of osteoarthritis (OA), and several pro- and anti-inflammatory cytokines have been implicated to mediate and regulate this process. Out of these cytokines, particularly IFNγ, IL-1β, IL-4 and IL-17 are associated with different phenotypes of T helper (T_H_) cells and macrophages, both examples of cells known for great phenotypic and functional heterogeneity. Chondrocytes also display various phenotypic changes during the course of arthritis. We set out to study the hypothesis of whether chondrocytes might adopt polarized phenotypes analogous to T_H_ cells and macrophages. We studied the effects of IFNγ, IL-1β, IL-4 and IL-17 on gene expression in OA chondrocytes with RNA-Seq. Chondrocytes were harvested from the cartilage of OA patients undergoing knee replacement surgery and then cultured with or without the cytokines for 24 h. Total RNA was isolated and sequenced, and GO (Gene Ontology) functional analysis was performed. We also separately investigated genes linked to OA in recent genome wide expression analysis (GWEA) studies. The expression of more than 2800 genes was significantly altered in chondrocytes treated with IL-1β [in the C(IL-1β) phenotype] with a fold change (FC) > 2.5 in either direction. These included a large number of genes associated with inflammation, cartilage degradation and attenuation of metabolic signaling. The profile of genes differentially affected by IFNγ (the C(IFNγ) phenotype) was relatively distinct from that of the C(IL-1β) phenotype and included several genes associated with antigen processing and presentation. The IL-17-induced C(IL-17) phenotype was characterized by the induction of a more limited set of proinflammatory factors compared to C(IL-1β) cells. The C(IL-4) phenotype induced by IL-4 displayed a differential expression of a rather small set of genes compared with control, primarily those associated with TGFβ signaling and the regulation of inflammation. In conclusion, our results show that OA chondrocytes can adopt diverse phenotypes partly analogously to T_H_ cells and macrophages. This phenotypic plasticity may play a role in the pathogenesis of arthritis and open new therapeutic avenues for the development of disease-modifying treatments for (osteo)arthritis.

## 1. Introduction

Osteoarthritis (OA) is the most common form of arthritis. It has been estimated to affect up to a half of the elderly population, and therefore causes widespread disability and human suffering as well as an immense burden to healthcare systems [1]. Once thought as a mostly mechanical “wear and tear” disease, the chronic inflammatory component of osteoarthritis has been increasingly recognized during recent decades [2]. Constant low-grade inflammation in the joint contributes to pain, oxidative stress, increased catabolism, and the eventual breakdown of articular cartilage [3,4]. Despite intense research, no disease-modifying pharmacological treatments are currently available for OA [5], demonstrating that our understanding of the pathogenesis of the disease remains limited.

When comparing chondrocytes from OA patients with healthy cells, several changes in gene expression can be observed [6,7]. The potential causal roles of these changes in the pathogenesis of OA are currently largely unknown. However, some of them can be considered harmful (such as secretion of catabolic enzymes and proinflammatory cytokines) and others protective (e.g., the production of extracellular matrix [ECM] components) [8,9]. The changes in OA chondrocyte phenotype are thought to be caused by several physical and chemical factors, among them local proinflammatory cytokines [10].

The T helper (T_H_) cell is probably the most well-known example of a cell capable of adopting distinct phenotypes in response to environmental factors. The different T_H_ phenotypes, in turn, are associated with different cytokines. The T_H_1 phenotype drives inflammation and defense against intracellular pathogens. These cells are induced by interleukin 12 (IL-12) and produce mainly interferon gamma (IFNγ) as an effector cytokine [11]. In addition, they induce macrophages to produce IL-1β, which in turn promotes the proinflammatory effects of T_H_1 cells [12]. T_H_2 cells are induced by interleukins 2 and 4. They secrete various factors that promote humoral immunity and regulate inflammation, of which IL-4 is regarded as the central cytokine [11]. T_H_17 cells are most closely associated with autoimmunity; they are induced by transforming growth factor beta (TGFβ) along with several proinflammatory cytokines, such as interleukins 6, 21 and 23, and they produce IL-17 as the central effector [13].

The macrophage is another cell type with well-defined differential phenotypes. The so-called “macrophage polarization” has two main phenotypes analogous to T_H_1 and T_H_2. The proinflammatory or “classically activated” M1 phenotype is associated with proinflammatory cytokines such as IL-1β and IFNγ, while the healing-promoting “alternatively activated” M2 phenotype is mainly linked to IL-4 [14]. The effects of IL-17 on macrophage phenotype have also attracted considerable interest. The M17 phenotype is not as well-defined as the M1 and M2 phenotypes; however, macrophages stimulated by IL-17 are characterized by the increased production of chemotactic and proinflammatory factors in the initial stages of the inflammatory response [15] and by the clearance of apoptotic cells and resolution of inflammation in the later phase [16].

Some authors have noted similarities between the variable functions and gene expression profiles of macrophages and chondrocytes in the setting of arthritis [17]. As another intriguing observation, major T_H_1/2/17 cytokines have been shown to play roles in the development of different forms of arthritis. Of the cytokines that have been implicated in the development of OA, IL-1β is probably the most prominent. It has been shown to decrease the anabolic activity in chondrocytes and promote their apoptosis [18]. It also induces the expression of the proteolytic enzymes of the matrix metalloproteinase (MMP) and a disintegrin-like and metalloproteinase with trombospondin motifs (ADAMTS) families [19]. OA chondrocytes have been shown to upregulate the expression of IL-1 receptor (IL-1R) increasing their sensitivity to this cytokine [20]. Despite this, systemic treatment strategies specifically targeting IL-1β seem to have rather limited efficacy in OA [21], and none have reached clinical use.

Another major proinflammatory cytokine playing a role in the pathogenesis of arthritis is interleukin 17A (IL-17A) [22]. It promotes inflammation in concert with other proinflammatory cytokines [23], and its concentration in the synovial fluid correlates with radiographic severity of joint destruction [24]. In chondrocytes, it induces proinflammatory and catabolic factors and reduces proteoglycan synthesis [25,26,27]. Along with other proinflammatory cytokines, it also increases bone degradation by activating RANK ligand (RANKL) in osteoclasts [28]. In a murine model of collagen-induced arthritis, IL-17 deficiency has been shown to protect joints from the disease and IL-17 overexpression to exacerbate it [29,30]. Some functional gene expression analyses have actually implicated IL-17 signaling as a pathophysiological factor over IL-1β, the cytokine long known to drive OA [31].

In contrast to IL-1β and IL-17, the potential role of IFNγ as a causative factor in OA has attracted less interest. However, it has been found to be upregulated in chondrocytes by proinflammatory cytokines [32] as well as to be present in OA synovial fluid [33]. Some gene variants that affect the development of OA, particularly those of T-cell immunoglobulin and mucin-domain containing-3 (TIM-3), exert their effects via the modulation of IFNγ expression [34].

In the light of the above connections between the cytokines linked to major T helper cell/macrophage phenotypes and OA, it can be hypothesized that chondrocytes might also adopt phenotypes analogous to T_H_1/2/17 or M1/2/17 cells, and that these phenotypes might play a role in the development of OA. In the present study, we investigated the effects of the central T_H_1/2/17 cytokines on gene expression in OA chondrocytes. We sought to identify significantly differentially expressed genes and modulated pathways. The results were also compared to those of a recent genome-wide association study comparing degraded OA cartilage to preserved cartilage [35]. To our knowledge, this is the first study comparing the effects of the central T_H_1/2/17 cytokines on OA chondrocytes and to characterize the resulting phenotypes.

## 2. Results

### 2.1. Effects of IL-1β on Chondrocyte Phenotype

After normalization and correction for multiple testing, a total of 2822 genes were found to be differentially expressed in IL-1β-treated chondrocytes [in the C(IL-1β) phenotype] versus controls in a statistically significant manner (FDR-corrected *p*-value < 0.05) and with a fold change (FC) 2.5 or more in either direction. Of these, 1092 were up- and 1730 downregulated. The list of the 20 most strongly upregulated genes contains several proinflammatory cyto- and chemokines, while the most strongly downregulated ones include several factors associated with regulation of gene expression, such as histone proteins (Table 1).

### 2.2. Effects of IL-17 on Chondrocyte Phenotype

Three hundred and eighty genes were differentially expressed in IL-17-treated chondrocytes [in the C(IL-17) phenotype] versus controls with FC > 2.5 in either direction, 314 of which were up- and 66 downregulated. Among the 20 most strongly upregulated genes were several associated with inflammation and chemotaxis, while the most strongly downregulated include genes involved in connective tissue development (Table 2).

### 2.3. Effects of IFNγ on Chondrocyte Phenotype

After normalization and correction for multiple testing, a total of 548 genes were found to be differentially expressed in IFNγ-treated chondrocytes [in the C(IFNγ) phenotype] versus controls in a statistically significant manner and FC 2.5 or more in either direction. Of these, 462 were up- and 86 downregulated. The 20 genes most strongly upregulated in C(IFNγ) cells included many associated with inflammation, antigen processing and presentation, and the regulation of proliferation. The most strongly downregulated genes included those involved in cell adhesion, proliferation and migration, and in Wnt signaling (Table 3).

### 2.4. Effects of IL-4 on Chondrocyte Phenotype

Twenty-six genes were upregulated by IL-4 with FC > 2.5 (Table S1). No genes were downregulated by IL-4 to a similar extent, but 10 genes were downregulated with FC < −1.5 (Table S2). In the C(IL-4) phenotype, the upregulated genes included those associated with the regulation of inflammation and TGFβ signaling as well as metabolism and cell adhesion, while several genes linked to cell proliferation were among the downregulated ones.

### 2.5. Functional Gene Categories in Different Chondrocyte Phenotypes

Table 4 shows the Gene Ontology (GO) terms affected with a high significance (FDR-corrected *p*-value < 0.01) by at least one studied proinflammatory cytokine (IL-1β, IFNγ or IL-17). The C(IL-1β) phenotype was involved in the activation of a wide range of inflammatory terms and pathways, along with those related to cell adhesion as well as extracellular matrix production and degradation. The T_H_17-associated cytokine IL-17 affected a partly overlapping, but smaller, set of inflammatory cytokines compared to IL-1β. The C(IFNγ) phenotype was quite distinct compared to the C(IL-1β) and C(IL-17) phenotypes; several terms related to antigen processing and presentation were affected by this cytokine alone. Nitric oxide synthase biosynthetic process and chemotaxis were among the functions involved solely in the C(IL-17) phenotype. In addition, many high-level GO terms related to inflammation were affected by all of the three proinflammatory cytokines.

In C(IL-4) cells, no significantly affected GO terms were detected when analyzing the genes with FC > 2.5 in either direction. When the FC threshold was lowered to 1.5, GO terms associated with cell division were among the significant ones (Table S3).

### 2.6. Comparing the Effects of Different Proinflammatory Cytokines

**Figure 1:**
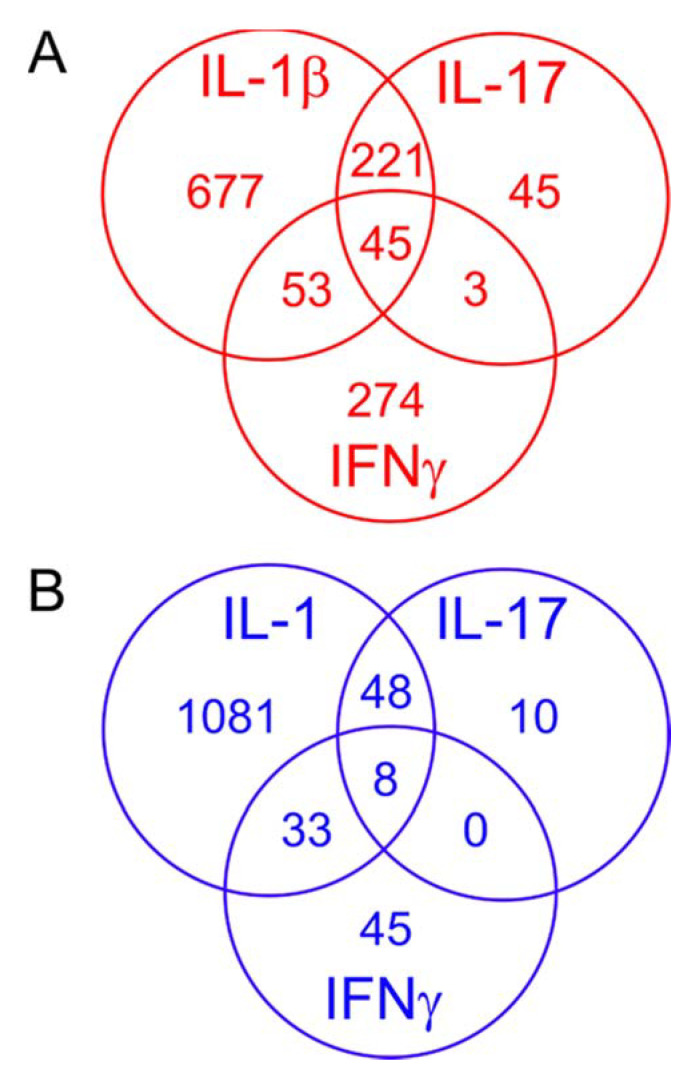
Venn diagrams of genes markedly upregulated (FC > 2.5) (**A**) or markedly downregulated (FC < 2.5) (**B**) by IL-1β, IL-17 and IFNγ. Red denotes up- and blue downregulated genes.

Next, we cross-compared the genes markedly upregulated (FC > 2.5) in the C(IL-1β), C(IFNγ) and C(IL-17) phenotypes to further characterize the differences and similarities between the resulting phenotypes. As shown in Figure 1A, a large portion (nearly 85%) of genes markedly upregulated in C(IL-17) cells were included in the large set of those similarly affected by IL-1β, but 45 genes were solely affected by IL-17, and the overlap of C(IL-17) and C(IFNγ) phenotypes was considerable smaller than that of C(IL-17) and C(IL-1β). The intersection of genes upregulated by both IL-17 and IFNγ was nearly completely contained in those upregulated by IL-1β (Figure 1A). Many central regulators of inflammation such as *IL6*, *PTGS2* (cyclo-oxygenase 2 or COX-2) and *NOS2* (inducible nitric oxide synthase or iNOS) were markedly upregulated by all the three T_H_1/T_H_17 cytokines, in line with the widespread activation of inflammatory pathways observed in the GO analysis (Table 5).

When comparing genes markedly downregulated (FC < −2.5) by the three proinflammatory cytokines, the large (>1000 genes) list of genes downregulated by IL-1β again contained a large proportion (85%) of those downregulated by IL-17 and a smaller amount (48%) of genes similarly affected by IFNγ (Figure 1B). Genes downregulated by all of the three cytokines are presented in Table 6 and include, for example, those associated with cell proliferation and skeletal system development.

### 2.7. Effects of the Cytokines on Genes Differentially Expressed in Degraded and Preserved OA Cartilage

Some previous studies have investigated the differences in gene expression between degraded and preserved OA cartilage. Of these, the study by Almeida et al. [35] is probably the most comprehensive. To see whether the studied cytokines shift chondrocyte phenotype towards either degraded or preserved cartilage, we compared the differentially expressed genes in the phenotypes observed in the present study to those differentially expressed in the study by Almeida et al. [35] As a very large number (over 2300) of significantly differentially expressed genes were identified in that study, we focused on those 84 genes which were most strongly upregulated (FC > 2.5 and FDR-corrected *p*-value < 0.01) in the degraded cartilage. Of those 84 genes, 38 were significantly affected by at least one of the proinflammatory cytokines (IL-1, IL-17 or IFNγ) in our data. A large majority (30) of these 38 genes were also upregulated by IL-1β, showing that the cytokine shifts chondrocyte phenotype towards the one observed in the degraded cartilage. Several mediators of inflammation, such as *LIF*, *CCL20* and *TREM1*, were especially strongly upregulated. Only four of the 84 genes (namely *CLIC3*, *ERFE*, *SLC27A2* and *ANK3*) were downregulated by IL-1β.

In the C(IFNγ) phenotype, 13 of the 84 genes associated with degraded cartilage (including *LIF* and *NGF*) were upregulated compared with control, but nearly as many (nine) were downregulated, including *TREM1*. This shows that the effects of IFNγ on chondrocyte phenotype in relation to the degraded/preserved cartilage are more ambiguous than those of IL-1β.

**Figure 2:**
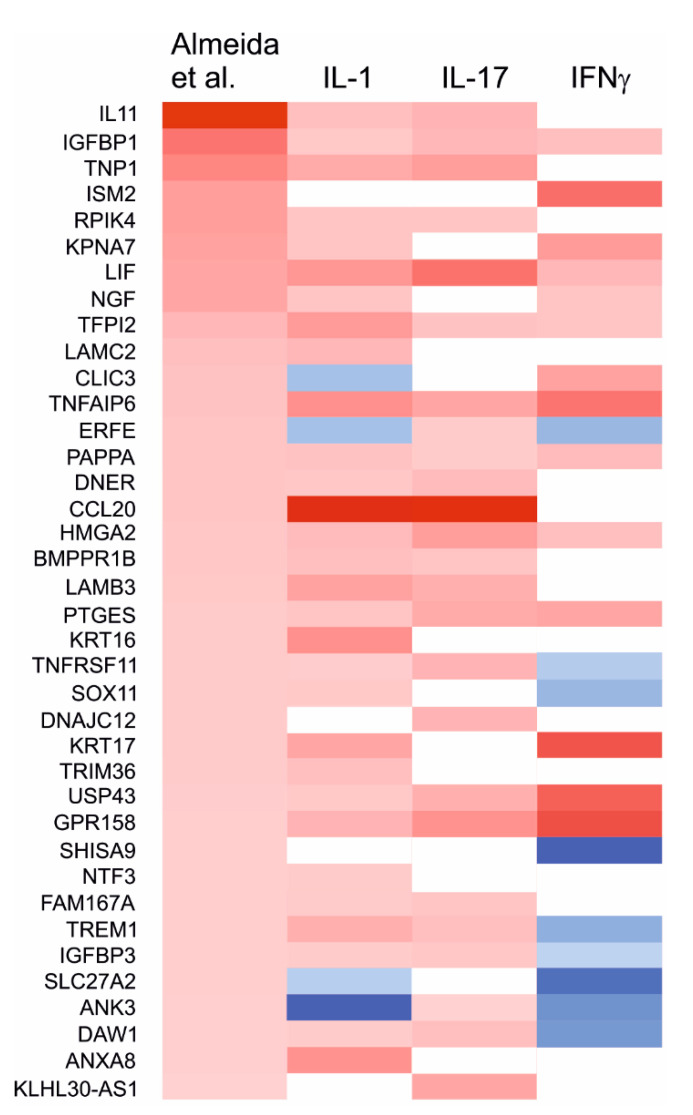
Heatmap of genes markedly upregulated (FC > 2.5) in degraded cartilage in the study by Almeida et al. [35] and significantly affected by at least one studied proinflammatory cytokine. Upregulated genes are marked with red, downregulated with blue, and genes with no significant fold change with white.

In C(IL-17) chondrocytes, 25 of the 84 genes associated with degraded cartilage were upregulated compared to naïve chondrocytes (including *CCL20* and *IL11*), and none were significantly downregulated. Nine genes, including *IGFBP1*, *LIF* and *GPR158*, were upregulated in all three inflammatory phenotypes C(IL-1β), C(IFNγ) and C(IL-17) and one (*ANK3*) was downregulated in all of them. (Figure 2 and Table S4).

**Figure 3:**
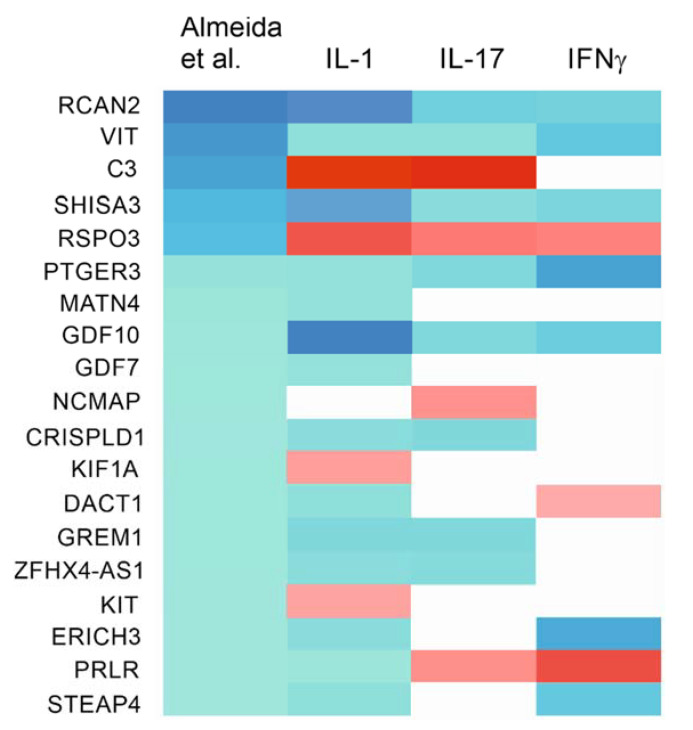
Heatmap of genes markedly downregulated (FC < −2.5) in degraded cartilage in the study by Almeida et al. [35] and significantly affected by at least one studied proinflammatory cytokine. Upregulated genes are marked with red, downregulated with blue, and genes with no significant fold change with white.

In the study by Almeida et al. [35], 52 genes were associated with preserved rather than degraded cartilage (i.e., significantly downregulated in degraded cartilage with FC < −2.5). Of these, 19 were significantly affected by at least one of the proinflammatory cytokines in our data. In C(IL-1β) cells, 13 of these 19 genes were significantly downregulated with *GDF10* displaying especially strong downregulation. In contrast, five of these genes were upregulated compared to control (including the especially strongly upregulated *C3* and *RSPO3*). This again shows that the net effect of IL-1β is to shift chondrocyte phenotype towards degraded cartilage. IFNγ showed a directionally similar, but less pronounced effect: seven of the genes associated with preserved cartilage were significantly downregulated and three upregulated in the C(IFNγ) phenotype. In C(IL-17) cells, eight genes associated with preserved cartilage were down- and four upregulated; *C3* once again displayed especially strong upregulation. Five genes, including *PTGER3* and *GDF10*, were downregulated in all of the three chondrocyte phenotypes. On the other hand, *RSPO3* and *PRLR*, both downregulated in degraded compared with preserved cartilage, were upregulated by all of the three cytokines. These data indicate that the C(IL-1β) and C(IL-17) phenotypes at least partly resemble the transcriptomic profile associated with degraded OA cartilage as identified by Almeida et al. [35]. In contrast, IFNγ seems to have a smaller effect on the genes directly linked to cartilage degradation in OA being instead characterized by the upregulation of genes associated with antigen processing and presentation. (Figure 3 and Table S5).

Relatively few genes were significantly affected by IL-4 in our data, and none of them were markedly (with FC > 2.5) associated with either degraded or preserved cartilage in the data of Almeida et al. [35]. However, looking at genes with a smaller proportional difference between degraded and preserved cartilage (FC > 1.5 in either direction) produced several genes that were significantly affected by IL-4. Ten genes (including *DUSP5* and *COL7A1*) were upregulated in degraded cartilage and also upregulated in C(IL-4) cells. In contrast, one gene associated with degraded cartilage (*HMMR*) was downregulated by IL-4, and seven genes (including *COL14A1*) associated with preserved cartilage were upregulated by IL-4. (Table S6)

To demonstrate that naïve chondrocytes can be affected by the cytokines studied, we separately studied the expression of their receptors. As shown in Table S7, receptors for all studied cytokines were expressed in unstimulated OA chondrocytes at meaningful levels.

## 3. Discussion

Chondrocytes from OA patients were found to adopt distinct phenotypes in response to the central T_H_1/T_H_2/T_H_17 cytokines. The phenotype induced by the T_H_1 cytokine interleukin 1 (IL-1β), the C(IL-1β) phenotype, can be characterized by widespread, strong upregulation of inflammation and catabolism as well as downregulation of metabolic signaling. The effects of the T_H_17 cytokine IL-17 appear to be somewhat less widespread and partly overlapping those of IL-1β, with induction of inflammatory and chemotactic factors. The phenotype induced by the second T_H_1 cytokine interferon gamma (IFNγ) seems to be distinct from both C(IL-1β) and C(IL-17) phenotypes, with a significant theme of antigen processing and presentation. The effects of the T_H_2 cytokine IL-4 were much more modest; some factors involved in the regulation of inflammation and TGFβ signaling were upregulated, while the downregulated genes were mostly associated with cell proliferation and migration.

In T cells, the T_H_1 phenotype drives inflammation and defense against intracellular pathogens (cell-mediated immunity) and is associated with the production of proinflammatory cytokines such as IFNγ and IL-1β [36]. Conversely, T_H_2 cells promote humoral immunity, regulate inflammation and direct resolving and injury-healing responses [11]. Central T_H_2 cytokines are IL-4 and IL-13. A third relatively well-established population of T_H_ cells is the T_H_17 phenotype. These cells produce IL-17, drive autoimmune reactions and activate neutrophils. This contrasts with T_H_1 cells that preferentially affect monocytes/macrophages, as well as T_H_2 cells that are associated with eosinophils, basophils and mast cells [37].

The central T_H_1/T_H_2/T_H_17 cytokines also induce loosely analogous macrophage phenotypes. Like T_H_1 cells, M1 or “classically activated” macrophages are induced by proinflammatory cytokines such as IL-1β and IFNγ and promote inflammation by secreting further proinflammatory factors. M2 or “alternatively activated” macrophages are induced canonically by IL-4. In addition to functioning as antiparasite effectors, they attenuate inflammation, direct wound-healing processes and promote the resolution of inflammation. [38] IL-17 induces a less-studied macrophage phenotype characterized by increased chemotaxis and the production of proinflammatory factors such as cyclo-oxygenase 2 (COX-2), IL-6 and tumor necrosis factor alpha (TNFα) [15,39] as well as resolution-promoting effects in the later phases of inflammation [16].

The chondrocyte phenotypes induced by different cytokines in our study can be considered analogous to T_H_ cell and particularly macrophage phenotypes. IL-1β affects a very large number of genes and induces a phenotype characterized by the expression of inflammatory and matrix-degrading genes. The C(IL-17) phenotype appears likewise proinflammatory, but with a somewhat more limited repertoire of inflammatory genes. C(IFNγ) also appears to be a phenotype that is inflammatory, but is also characterized by genes linked to antigen presentation. The C(IL-4) phenotype is characterized by the expression of genes linked to TGFβ signaling and the regulation of inflammation.

The chondrocyte phenotypes induced by the T_H_1/T_H_2/T_H_17 cytokines appeared to be quite distinct as only 45 genes were markedly (FC > 2.5) upregulated and eight markedly downregulated (FC < −2.5) by all three proinflammatory cytokines, considering that hundreds of genes were up- and dozens downregulated to a similar extent by each of the three cytokines. The factors upregulated by all of the three proinflammatory cytokines (IL-1β, IFNy and IL-17) include the well-known inflammatory mediators *IL6*, nitric oxide synthase 2/inducible nitric oxide synthase (*NOS2*/iNOS) and prostaglandin-endoperoxide synthase 2/cyclooxygenase 2 (*PTGS2*/COX-2). On this list were also included, for example, pentraxin 3 (*PTX3*), toll-like receptor 2 (*TLR2*), chemokine (C-C motif) ligand 2 (*CCL2*), interferon regulatory factor 4 (*IRF4*) and prolactin receptor (*PRLR*). Pentraxin 3 (*PTX*) promotes inflammation by activating the classical complement pathway and by facilitating antigen recognition by mononuclear phagocytes [40], and it has been shown to be elevated in the serum and synovial fluid of patients with rheumatoid arthritis [41]. *TLR2* is a pattern recognition receptor mediating innate immune activation by microbial particles. In osteoarthritis, it is activated by hyaluronan and aggrecan fragments leading to the activation of nuclear factor kappa-light-chain-enhancer of activated B cells (NF-κB) signaling, which may contribute to OA progression and pain [42,43]. *CCL2* is a monocyte-attracting chemokine that has been linked to OA development and pain [44,45]. *IRF4* has recently been associated with cartilage destruction and pain in OA via the induction of CCL17 [46]. Prolactin has been implicated to promote chondrocyte differentiation and attenuate apoptosis, and thus the upregulation of its receptor might promote cartilage survival [47,48].

Factors downregulated by all of the three proinflammatory cytokines include asporin (*ASPN*) and prostaglandin EP3 receptor (*PTGER3*). Asporin belongs to the family of leucine-rich repeat proteins and is associated with cartilage matrix, also bearing a similarity to decorin [49]. The potential role of asporin in OA appears to be unclear; several studies have linked the protein to the development of the disease, where it might impair chondrogenesis by inhibiting TGF-β signaling [50]. Polymorphisms of the asporin gene have also been linked to OA risk [51], even though the most recent meta-analysis failed to find evidence for this [52]. Prostaglandin E2 (PGE2)-induced *PTGER3* downregulation may contribute to cartilage inflammation and damage via NF-κB activation and IL-6 synthesis [53].

When the Gene Ontology (GO) terms significantly affected by the three different proinflammatory cytokines were studied, all three were found to affect those associated with inflammation. IL-1β was alone in significantly affecting several terms, such as cell adhesion, extracellular matrix metabolism and collagen catabolism, linking the chondrocyte phenotype induced by this cytokine to these functions. IL-17 solely affected nitric oxide synthase biosynthesis. This is intriguing, as the nitric oxide production is an important part of inflammatory response in chondrocytes [54]. The C(IFNγ) phenotype seems to be differentiated from others by activation of pathways related to antigen processing and presentation. Chondrocytes are not considered “professional” antigen-presenting cells, but they have, interestingly, been shown to present cartilage proteoglycans as antigens to CD8+ T cells, potentially contributing to local joint inflammation [55,56].

Previously published genome-wide expression analyses (GWEAs) have identified a number of differentially expressed genes between either damaged and intact OA cartilage or healthy and OA cartilage. These include genes involved in inflammation, skeletal system development, cell adhesion and monosaccharide metabolism [35,57,58,59]. When comparing our results to those of the comprehensive study by Almeida et al. [35], the C(IL-1β) phenotype most closely resembled degraded OA cartilage, while IL-17 upregulated a smaller number of proinflammatory factors associated with degraded cartilage in that study. Accordingly, some genes associated with preserved as opposed to degraded cartilage were also downregulated by these proinflammatory cytokines. Most of these genes are linked to cartilage anabolism. The effects of IFNγ and (especially) IL-4 on the genes identified by Almeida et al. [35] were more modest. It is important to note that the receptors for all cytokines studied were expressed at marked levels in our samples, which lends further validity to our results.

A potential limitation of the study is that whole thickness pieces of cartilage obtained from joint replacement surgery were used for chondrocyte isolation. Thus, the cells obtained are likely a mixture of chondrocytes from different layers of cartilage, and there might be some differences in the effects of cytokines between these groups. However, all chondrocytes can be expected to be exposed to cytokines diffused from the synovial fluid and/or produced by chondrocytes (in autocrine or paracrine manner). Thus, we think that the observed clear differences in the chondrocyte phenotypes in response to the major T_H_1/T_H_2/T_H_17 cytokines are relevant for further understanding of chondrocyte biology and OA pathophysiology. In future studies, cartilage layer-specific cell isolation methods or single-cell RNA-Seq could be considered to unravel possible zone-specific responses.

Another limitation of the study is that the chondrocytes used were obtained from OA joints; therefore, some of the detected effects of the cytokines might differ from those observed in healthy chondrocytes. Studying the effects of the cytokines on healthy chondrocytes would be an interesting avenue of future study; however, obtaining healthy primary human chondrocytes presents a practical challenge (compared to OA chondrocytes which can be obtained from joint replacement surgery). In the present study, we observed similarities between the C(IL-1β) and C(Il-17) phenotypes and the gene expression profile of chondrocytes from degraded OA cartilage published by Almeida et al. [35]; C(IFNγ) and especially C(IL-4) bore less resemblance to that phenotype. This suggests that the cytokine-induced phenotypes observed in our data have relevance regarding OA pathogenesis.

In conclusion, OA chondrocytes, analogously to macrophages, can assume distinct phenotypes in response to the cytokines associated with the T_H_1/T_H_2/T_H_17 phenotypes of T helper cells. These results provide novel information on chondrocyte biology and the pathogenesis of OA with further insights into the development of disease-modifying drugs for (osteo)arthritis.

## 4. Materials and Methods

### 4.1. Cartilage and Cell Culture

Leftover cartilage pieces were collected from nine patients undergoing total knee replacement surgery in Coxa Hospital for Joint Replacement, Tampere, Finland. All patients fulfilled the American College of Rheumatology classification criteria for knee OA [60]. Patients with diabetes mellitus were excluded from the study to avoid potential confounding effects on chondrocyte metabolism [61]. The study was approved by the Ethics Committee of Tampere University Hospital, Finland, and carried out in accordance with the Declaration of Helsinki. Written informed consent was obtained from the patients. Chondrocyte isolation and culture was carried out as previously described [62]. To ensure an adequate yield of chondrocytes, all available cartilage was removed aseptically using a scalpel from the bony parts received from joint replacement surgery and cut into small pieces. The pieces were first washed with phosphate buffered saline (PBS). After that, they were incubated for 24 h in the presence of Liberase enzyme (Roche, Mannheim, Germany) 0.25 mg/mL, diluted in serumless Dulbecco’s modified Eagle’s medium (DMEM, Sigma-Aldrich, St Louis, MO, USA) with glutamax-I containing penicillin (100 units/mL), streptomycin (100 μg/mL), and amphotericin B (250 ng/mL) (all three from Invitrogen, Carlsbad, CA, USA) at 37 °C. The resulting cell suspension was poured through a 70 μm nylon mesh and centrifuged for five minutes at 200 g. Cells were then washed twice and seeded on 24-well plates (0.2 million cells/mL) in DMEM supplemented with 10% heat-inactivated fetal bovine serum (Lonza) together with the aforementioned compounds. Confluent cultures were exposed to fresh culture medium alone, with 10 ng/mL IFNγ, with 100 pg/mL IL-1β, with 50 ng/mL IL-17 or with 10 ng/mL IL-4, for 24 h. The concentrations used were chosen based on our preliminary experiments with cultured chondrocytes.

### 4.2. RNA Isolation and Sample Preparation

Culture medium was removed at the indicated time points and total RNA of the chondrocytes was extracted with GenElute Mammalian Total RNA Miniprep kit (Sigma-Aldrich). The sample was treated with DNAse I (Fermentas UAB, Vilnius, Lithuania). RNA concentration and integrity were confirmed with the 2100 Bioanalyzer (Agilent Technologies, Santa Clara, CA, USA).

### 4.3. Next Generation Sequencing and Data Analysis

Sequencing of samples was performed in the Finnish Institute of Molecular Medicine (FIMM) sequencing core, Helsinki, Finland, using the Illumina HiSeq 2500 sequencing platform. Sequencing depth was 20 million paired-end reads 100 bp in length. Read quality was first assessed using FastQC [63], and the reads were trimmed using Trimmomatic [64]. Trimmed reads were aligned to reference human genome with STAR [65]. Count matrices were prepared with the featureCounts program [66]. Differential expression was assessed with DESeq2 [67]. Gene expression levels were given as DeSeq2-normalized counts, and genes with an average normalized count 10 or less across all samples were excluded from further analysis. For the purposes of further analysis, genes with a minimum of 2.5 fold change (FC) in abundance and FDR-corrected *p*-value < 0.05 were deemed biologically and statistically significant (unless otherwise indicated). Functional analysis was performed against the Gene Ontology (GO) database [68,69] using the DAVID tool [70], and REVIGO was used to reduce the resulting list [71].

### 4.4. Statistics

For NGS data analysis, normalization was performed and differential expression studied using a negative binomial model implemented in DESeq2.

## Figures and Tables

**Table 1:** Twenty most strongly up- and downregulated genes in interleukin 1-treated OA chondrocytes (IL1) relative to control (Co).

Gene	Name	Function	Mean (Co)	Mean (IL1)	Fold Change	adj. p
*IL6*	Interleukin 6	Inflammation	12.4	18,406.9	**3685.72**	<1.0 × 10^−4^
*CXCL1*	C-X-C motif chemokine ligand 1	Inflammation, chemotaxis	13.8	23,793.7	**3457.68**	<1.0 × 10^−4^
*IL1B*	Interleukin 1 beta	Inflammation	2.8	9575.7	**3332.44**	<1.0 × 10^−4^
*CXCL8*	C-X-C motif chemokine ligand 8	Inflammation, chemotaxis	329.5	855,146.3	**2968.9**	<1.0 × 10^−4^
*CXCL6*	C-X-C motif chemokine ligand 6	Inflammation, chemotaxis	2.8	4951.8	**2352.02**	<1.0 × 10^−4^
*CXCL5*	C-X-C motif chemokine ligand 5	Inflammation, chemotaxis	7.4	7352.4	**1239.8**	<1.0 × 10^−4^
*CXCL2*	C-X-C motif chemokine ligand 2	Inflammation, chemotaxis	3.9	4798.2	**1198.05**	<1.0 × 10^−4^
*CXCL3*	C-X-C motif chemokine ligand 3	Inflammation, chemotaxis	3.1	3154.6	**1130.76**	<1.0 × 10^−4^
*CCL20*	C-C motif chemokine ligand 20	Inflammation, chemotaxis	418	381,100.8	**1128.35**	<1.0 × 10^−4^
*IL36RN*	Interleukin 36 receptor antagonist	Regulation of inflammation	8.6	5863.8	**914.19**	<1.0 × 10^−4^
*ADORA2A*	Adenosine A2a receptor	Regulation of inflammation	5.5	1550.7	**641.44**	<1.0 × 10^−4^
*IL36G*	Interleukin 36 gamma	Inflammation	1.8	1065.5	**562.03**	<1.0 × 10^−4^
*EREG*	Epiregulin	Regulation of proliferation	31.9	13,697.7	**506.87**	<1.0 × 10^−4^
*CSF3*	Colony stimulating factor 3	Granulocyte-mediated inflammation	0.1	63.9	**300.02**	<1.0 × 10^−4^
*VNN1*	Vanin 1	T cell migration	9.2	2467.2	**273.35**	<1.0 × 10^−4^
*CCL5*	C-C motif chemokine ligand 5	Inflammation, chemotaxis	4.1	1134.2	**271.85**	<1.0 × 10^−4^
*C15orf48*	Chromosome 15 open reading frame 48	?	27.2	4669.1	**253.13**	<1.0 × 10^−4^
*CCL3*	C-C motif chemokine ligand 3	Inflammation, granulocyte activation	0.5	166.3	**242.88**	<1.0 × 10^−4^
*FCAMR*	Fc fragment of IgA and IgM receptor	Adaptive immunity, leukocyte migration	2.6	492	**213.45**	<1.0 × 10^−4^
*SERPINB7*	Serpin family B member 7	Endoproteinase inhibition	22.1	3747.9	**205.63**	<1.0 × 10^−4^
*HRCT1*	Histidine rich carboxyl terminus 1	?	105.8	4.1	**−38.85**	<1.0 × 10^−4^
*LSP1*	Lymphocyte specific protein 1	Regulation of neutrophil mobility	1749.6	58.1	**−31.39**	<1.0 × 10^−4^
*HIST1H3G*	Histone cluster 1 H3 family member g	Regulation of transcription	183.4	9.6	**−28.26**	<1.0 × 10^−4^
*ACTC1*	Actin, alpha, cardiac muscle 1	Heart muscle constituent	195.2	10.5	**−24.79**	<1.0 × 10^−4^
*NXPH3*	Neurexophilin 3	?	39.2	2.4	**−23.89**	<1.0 × 10^−4^
*SCN2B*	Sodium voltage-gated channel beta subunit 2	Cell adhesion and migration	167	8.7	**−22.19**	<1.0 × 10^−4^
*HIST1H1A*	Histone cluster 1 H1 family member a	?	908.5	47.2	**−21.2**	<1.0 × 10^−4^
*GDF10*	Growth differentiation factor 10	Skeletal system development	813.6	45.7	**−20.57**	<1.0 × 10^−4^
*LINC02593*	Long intergenic non-protein coding RNA 2593	?	68.3	3.4	**−20.53**	<1.0 × 10^−4^
*HIST1H3B*	Histone cluster 1 H3 family member b	Regulation of transcription	990.6	59.2	**−20.46**	<1.0 × 10^−4^
*TMEM26*	Transmembrane protein 26	?	403.7	21.4	**−19.3**	<1.0 × 10^−4^
*PHYHIPL*	Phytanoyl-CoA 2-hydroxylase interacting protein like	?	22	1.6	**−19.19**	<1.0 × 10^−4^
*SARDH*	Sarcosine dehydrogenase	Mitochondrial metabolism	25.8	2.4	**−19.08**	<1.0 × 10^−4^
*HIST1H2BO*	Histone cluster 1 H2B family member o	Regulation of transcription?	234.4	12.7	**−18.99**	<1.0 × 10^−4^
*ID3*	Inhibitor of DNA binding 3, HLH protein	Regulation of transcription	676.5	45.8	**−18.32**	<1.0 × 10^−4^
*HIST1H2AJ*	Histone cluster 1 H2A family member j	Regulation of transcription?	857	47.1	**−18.12**	<1.0 × 10^−4^
*HIST1H1B*	Histone cluster 1 H1 family member b	Regulation of transcription?	736	50.6	**−17.69**	<1.0 × 10^−4^
*MFAP2*	Microfibril associated protein 2	ECM organization	33	3.2	**−17.52**	<1.0 × 10^−4^
*TNNT3*	Troponin T3, fast skeletal type	Muscle constituent	95.6	6.4	**−17.51**	<1.0 × 10^−4^
*HIST1H2AL*	Histone cluster 1 H2A family member l	Regulation of transcription?	321.4	21.2	**−17.32**	<1.0 × 10^−4^

Red = upregulated genes; blue = downregulated genes.

**Table 2:** Twenty most strongly up- and downregulated genes in interleukin 17-treated OA chondrocytes (IL17) relative to control (Co).

Gene	Name	Function	Mean (Co)	Mean (IL17)	Fold Change	adj. p
*SAA2*	Serum amyloid A2	Chemotaxis	5.5	659.2	**319.99**	<1.0 × 10^−4^
*IL6*	Interleukin 6	Inflammation	12.2	1431.4	**250.15**	<1.0 × 10^−4^
*SAA1*	Serum amyloid A1	Inflammation, chemotaxis	63.7	3520.0	**183.26**	<1.0 × 10^−4^
*SAA2-SAA4*	SAA2-SAA4 readthrough	Chemotaxis?	2.9	216.7	**156.18**	<1.0 × 10^−4^
*CXCL6*	C-X-C motif chemokine ligand 6	Inflammation, chemotaxis	2.8	276.4	**141.01**	<1.0 × 10^−4^
*CXCL1*	C-X-C motif chemokine ligand 1	Inflammation, chemotaxis	13.6	1170.5	**136.48**	<1.0 × 10^−4^
*VNN1*	Vanin 1	T cell migration	9.1	820.5	**84.13**	<1.0 × 10^−4^
*CCL20*	C-C motif chemokine ligand 20	Chemotaxis	412.8	26,508.9	**73.49**	<1.0 × 10^−4^
*TNFSF18*	TNF superfamily member 18	T cell survival	4.2	470.3	**73.05**	<1.0 × 10^−4^
*IL36RN*	Interleukin 36 receptor antagonist	Regulation of inflammation	8.5	468.0	**69.09**	<1.0 × 10^−4^
*VNN3*	Vanin 3	?	1.8	130.3	**66.35**	<1.0 × 10^−4^
*ADORA2A*	Adenosine A2a receptor	Inflammation, phagocytosis	5.4	105.9	**64.74**	<1.0 × 10^−4^
*CXCL2*	C-X-C motif chemokine ligand 2	Inflammation, chemotaxis	3.9	220.3	**55.90**	<1.0 × 10^−4^
*CXCL8*	C-X-C motif chemokine ligand 8	Inflammation, chemotaxis	324.8	14,116.5	**48.18**	<1.0 × 10^−4^
*C15orf48*	Chromosome 15 open reading frame 48	Mitochondrial respiration?	26.9	820.3	**46.34**	<1.0 × 10^−4^
*PDZK1IP1*	PDZK1 interacting protein 1	Regulation of apoptosis	5.2	206.9	**41.18**	<1.0 × 10^−4^
*NOS2*	Nitric oxide synthase 2	Inflammation	137.9	3370.2	**40.02**	<1.0 × 10^−4^
*ODAPH*	Odontogenesis associated phosphoprotein	Enamel production	1.4	41.9	**37.29**	<1.0 × 10^−4^
*SLC28A3*	Solute carrier family 28 member 3	Nucleoside transport	4.3	150.4	**35.34**	<1.0 × 10^−4^
*CXCL5*	C-X-C motif chemokine ligand 5	Inflammation, chemotaxis	7.3	207.5	**34.25**	<1.0 × 10^−4^
*ACTC1*	Actin, alpha, cardiac muscle 1	Cardiac muscle component	191.7	26.7	**−8.14**	<1.0 × 10^−4^
*TOX*	Thymocyte selection associated high mobility group box	T cell development	14.6	3.9	**−5.66**	0.0010
*TMEM26*	Transmembrane protein 26	?	396.3	69.8	**−5.47**	<1.0 × 10^−4^
*TNNT3*	Troponin T3, fast skeletal type	Muscle component	93.9	17.9	**−5.28**	<1.0 × 10^−4^
*TENT5B*	Terminal nucleotidyltransferase 5B	Regulation of cell proliferation	152.5	39.7	**−4.81**	<1.0 × 10^−4^
*TMEM26-AS1*	TMEM26 antisense RNA 1	?	32.0	14.4	**−4.77**	3.8 × 10^−4^
*RCAN2*	Regulator of calcineurin 2	Regulation of transcription	326.5	74.6	**−4.74**	<1.0 × 10^−4^
*OPRL1*	Opioid related nociceptin receptor 1	?	11.8	3.0	**−4.51**	0.0068
*CSRNP3*	Cysteine and serine rich nuclear protein 3	Regulation of apoptosis	59.7	19.7	**−4.01**	<1.0 × 10^−4^
*ASPN*	Asporin	Cartilage constituent	2011.2	505.2	**−3.92**	<1.0 × 10^−4^
*HRCT1*	Histidine rich carboxyl terminus 1	?	104.1	25.8	**−3.85**	<1.0 × 10−4
*AQP1*	Aquaporin 1 (Colton blood group)	Regulation of osmotic pressure, angiogenesis, apoptosis	42.9	13.4	**−3.69**	<1.0 × 10^−4^
*YWHAZP5*	YWHAZ pseudogene 5	?	10.2	3.2	**−3.68**	0.013
*MRAP2*	Melanocortin 2 receptor accessory protein 2	cAMP signaling	1295.9	376.5	**−3.62**	<1.0 × 10^−4^
*C1QTNF7*	C1q and TNF related 7	?	63.4	20.1	**−3.54**	<1.0 × 10^−4^
*MFAP2*	Microfibril associated protein 2	Connective tissue organization	32.4	8.7	**−3.47**	<1.0 × 10^−4^
*CLEC3A*	C-type lectin domain family 3 member A	Skeletal system development	847.3	264.6	**−3.46**	<1.0 × 10^−4^
*GREM1*	Gremlin 1, DAN family BMP antagonist	Regulation of connective tissue development	5141.6	1566.4	**−3.41**	<1.0 × 10^−4^
*CRISPLD1*	Cysteine rich secretory protein LCCL domain containing 1	Morphogenesis	946.1	280.2	**−3.39**	<1.0 × 10^−4^
*HRASLS5 (=PLAAT5)*	HRAS like suppressor family member 5	Glycerophospholipid metabolism	12.8	3.6	**−3.37**	0.019

Red = upregulated genes; blue = downregulated genes.

**Table 3:** Twenty most strongly up- and downregulated genes in interferon gamma -treated OA chondrocytes (IFNγ) relative to control (Co).

Gene	Name	Function	Mean (Co)	Mean (IFNγ)	Fold change	adj. p
*IDO1*	Indoleamine 2,3-dioxygenase 1	Regulation of T cell -mediated immunity	17.5	42,320.0	**4643.74**	<1.0 × 10^−4^
*LGALS17A*	Galectin 14 pseudogene	?	0.4	1065.1	**1750.58**	<1.0 × 10^−4^
*GBP1P1*	Guanylate binding protein 1 pseudogene 1	?	2.6	2838.8	**1245.34**	<1.0 × 10^−4^
*CXCL10*	C-X-C motif chemokine ligand 10	Chemotaxis	2.2	2065.2	**1117.91**	<1.0 × 10^−4^
*GBP5*	Guanylate binding protein 5	Inflammasome activation	1.4	1518.3	**1112.44**	<1.0 × 10^−4^
*CXCL9*	C-X-C motif chemokine ligand 9	T cell chemotaxis	1.1	1069.9	**1033.80**	<1.0 × 10^−4^
*GBP4*	Guanylate binding protein 4	Inflammation?	30.9	27,565.6	**955.57**	<1.0 × 10^−4^
*IFI44L*	Interferon induced protein 44 like	?	9.7	6185.8	**694.66**	<1.0 × 10^−4^
*GBP1*	Guanylate binding protein 1	Negative regulation of inflammation	124.3	54,562.1	**454.62**	<1.0 × 10^−4^
*HLA-DRA*	Major histocompatibility complex, class II, DR alpha	Antigen presentation	5.6	2338.3	**408.93**	<1.0 × 10^−4^
*HLA-DRB1*	Major histocompatibility complex, class II, DR beta 1	Antigen presentation	10.7	2430.7	**383.18**	<1.0 × 10^−4^
*CD74*	CD74 molecule	Antigen presentation	31.9	11,211.5	**353.35**	<1.0 × 10^−4^
*RSAD2*	Radical S-adenosyl methionine domain containing 2	Antiviral action	44.5	15,365.2	**338.82**	<1.0 × 10^−4^
*RARRES3*	Retinoic acid receptor responder 3	Phospholipid catabolism	33.1	8271.1	**286.40**	<1.0 × 10^−4^
*BST2*	Bone marrow stromal cell antigen 2	Antiviral action	10.1	2908.5	**285.04**	<1.0 × 10^−4^
*GBP6*	Guanylate binding protein family member 6	Inflammation	1.0	193.3	**273.26**	<1.0 × 10^−4^
*HLA-DRB5*	Major histocompatibility complex, class II, DR beta 5	Antigen presentation	4.4	825.4	**253.47**	<1.0 × 10^−4^
*HLA-DRB6*	Major histocompatibility complex, class II, DR beta 6 (pseudogene)	Antigen presentation?	0.3	125.7	**226.68**	<1.0 × 10^−4^
*APOL4*	Apolipoprotein L4	Lipid metabolism	2.6	500.8	**225.95**	<1.0 × 10^−4^
*IFIT2*	Interferon induced protein with tetratricopeptide repeats 2	Regulation of proliferation	96.2	20,648.8	**225.79**	<1.0 × 10^−4^
*TNFRSF10D*	TNF receptor superfamily member 10d	Inhibition of apoptosis	4135.1	501.9	**−7.65**	<1.0 × 10^−4^
*ARHGAP9*	Rho gtpase activating protein 9	?	10.7	2.4	**−5.27**	0.0028
*NANOS1*	Nanos C2HC-type zinc finger 1	Regulation of translation and cell migration	83.4	16.9	**−4.94**	<1.0 × 10^−4^
*SNORD108*	Small nucleolar RNA, C/D box 108	?	66.6	13.8	**−4.81**	<1.0 × 10^−4^
*FAM189A2*	Family with sequence similarity 189 member A2	?	13.6	4.3	**−4.39**	0.0033
*PWAR6*	Prader Willi/Angelman region RNA 6	?	34.0	7.9	**−4.32**	<1.0 × 10^−4^
*GABRA4*	Gamma-aminobutyric acid type A receptor alpha4 subunit	Synaptic transmission	2346.1	549.2	**−4.28**	<1.0 × 10^−4^
*CORO2A*	Coronin 2A	?	13.5	3.7	**−4.11**	0.020
*WFDC1*	WAP four-disulfide core domain 1	Regulation of proliferation	65.1	18.0	**−4.06**	<1.0 × 10^−4^
*PRSS35*	Serine protease 35	?	51.4	13.5	**−4.01**	<1.0 × 10^−4^
*SLC16A14*	Solute carrier family 16 member 14	Organic acid transport	40.2	13.3	**−3.98**	<1.0 × 10^−4^
*PWAR5*	Prader Willi/Angelman region RNA 5	?	359.7	91.4	**−3.93**	<1.0 × 10^−4^
*MTURN*	Maturin, neural progenitor differentiation regulator homolog	?	1857.1	519.7	**−3.63**	<1.0 × 10^−4^
*C1QTNF5*	C1q and TNF related 5	Cell adhesion	152.4	46.1	**−3.47**	<1.0 × 10^−4^
*LONRF2*	LON peptidase N-terminal domain and ring finger 2	?	206.8	59.5	**−3.46**	<1.0 × 10^−4^
*FGFR4*	Fibroblast growth factor receptor 4	Cell proliferation and migration	11.1	5.1	**−3.31**	0.045
*TRABD2B*	Trab domain containing 2B	Wnt signaling, proteolysis	14.2	5.5	**−3.29**	0.0014
*TNNT3*	Troponin T3, fast skeletal type	Muscle contraction	106.0	31.6	**−3.26**	<1.0 × 10^−4^
*NCALD*	Neurocalcin delta	Endocytosis	17.3	6.6	**−3.24**	0.029
*CDH2*	Cadherin 2	Cell adhesion	12.0	4.1	**−3.23**	0.0012

Red = upregulated genes; blue = downregulated genes.

**Table 4:** GO terms affected by different proinflammatory cytokines. Genes with FC > 2.5 in either direction were analyzed with DAVID, and the resulting lists were reduced with REVIGO. GO terms significantly affected (with FDR-corrected *p*-value < 0.05) by a cytokine are marked with an X.

Term	IL1	IL17	IFNγ	Term	IL1	IL17	IFNγ
Inflammatory response	**X**	**X**	**X**	Nucleosome assembly	**X**		
Immune response	**X**	**X**	**X**	Chromosome segregation	**X**		
Response to lipopolysaccharide	**X**	**X**	**X**	Protein heterotetramerization	**X**		
Chemotaxis	**X**	**X**	**X**	Wound healing	**X**		
Negative regulation of viral entry	**X**	**X**	**X**	Regulation of cell proliferation	**X**		
into host cell				Cell migration	**X**		
Negative regulation of type I	**X**	**X**	**X**	Regulation of gene silencing	**X**		
interferon production				Positive regulation of interleukin-12 production	**X**		
Response to progesterone	**X**	**X**		Odontogenesis	**X**		
Cell-cell signaling	**X**	**X**		Cellular response to mechanical stimulus	**X**		
Angiogenesis	**X**	**X**		Peptidyl-tyrosine phosphorylation	**X**		
Negative regulation of growth	**X**	**X**		Collagen catabolic process	**X**		
Positive regulation of mitotic	**X**	**X**		Positive regulation of cell division	**X**		
nuclear division				Positive chemotaxis		**X**	
Negative regulation of cell	**X**	**X**		Positive regulation of nitric-oxide synthase biosynthetic		**X**	
proliferation				process			
Signal transduction	**X**		**X**	Acute-phase response		**X**	
Response to virus	**X**		**X**	Positive regulation of cytosolic calcium ion concentration		**X**	
Positive regulation of interleukin-6	**X**		**X**	Positive regulation of gtpase activity			**X**
production				Response to glucocorticoid			**X**
Response to hydrogen peroxide	**X**		**X**	Response to wounding			**X**
Positive regulation of I-kappab	**X**		**X**	Positive regulation of NF-kappab transcription factor			**X**
kinase/NF-kappab signaling				activity			
Response to drug	**X**		**X**	Negative regulation of tumor necrosis factor production			**X**
Cellular response to zinc ion		**X**	**X**	Cellular response to organic cyclic compound			**X**
Response to toxic substance		**X**	**X**	Antigen processing and presentation			**X**
Tumor necrosis factor-mediated		**X**	**X**	Antigen processing and presentation of peptide or			**X**
signaling pathway				polysaccharide antigen via MHC class II			
Cell division	**X**			Antigen processing and presentation of exogenous peptide			**X**
DNA replication	**X**			antigen via MHC class I, TAP-independent			
Telomere organization	**X**			Response to interferon-beta			**X**
Positive regulation of gene	**X**			Response to interferon-alpha			**X**
expression				T cell costimulation			**X**
Cell adhesion	**X**			Positive regulation of T cell mediated cytotoxicity			**X**
Extracellular matrix organization	**X**			Defense response			**X**
Skeletal system development	**X**			Protein trimerization			**X**
Sister chromatid cohesion	**X**			Proteolysis			**X**
DNA replication initiation	**X**			Defense response to protozoan			**X**
Cellular protein metabolic process	**X**			Positive regulation of peptidyl-tyrosine phosphorylation			**X**
Cell proliferation	**X**			Protein polyubiquitination			**X**
Negative regulation of gene	**X**						
expression, epigenetic							

**Table 5:** Genes upregulated by all studied proinflammatory cytokines with FC > 2.5. Shown are mean normalized expression levels in control (Co) and in C(IL1), C(IL17) and C(IFNγ) phenotypes, fold changes (FCs) for all comparisons vs. control and false discovery rate (FDR)-adjusted *p* values for them.

Gene	Name	Mean exp. (Co)	Mean exp. (IL1)	Mean exp. (IL17)	Mean exp. (IFNγ)	FC (IL1 vs. Co)	adj. p (IL1 vs. Co)	FC (IL17 vs. Co)	adj. p (IL17 vs. Co)	FC (IFNγ vs. Co)	adj. p (IFNγ vs. Co)
*IL6*	Interleukin 6	12.8	18,406.9	1431.4	94.2	**3685.72**	<1.0 × 10^−4^	**250.15**	<1.0 × 10^−4^	**12.34**	<1.0 × 10^−4^
*IL36RN*	Interleukin 36 receptor antagonist	8.9	5863.8	468.0	36.7	**914.19**	<1.0 × 10^−4^	**69.09**	<1.0 × 10^−4^	**4.59**	<1.0 × 10^−4^
*ESM1*	Endothelial cell specific molecule 1	276.7	37,984.1	1373.5	1449.2	**157.25**	<1.0 × 10^−4^	**5.09**	<1.0 × 10^−4^	**4.70**	<1.0 × 10^−4^
*SAA2*	Serum amyloid A2	5.8	371.4	659.2	27.1	**149.11**	<1.0 × 10^−4^	**319.99**	<1.0 × 10^−4^	**8.73**	<1.0 × 10^−4^
*iNOS/NOS2*	Inducible nitric oxide synthase/Nitric oxide synthase 2	144.2	12,704.9	3370.2	3046.1	**131.22**	<1.0 × 10^−4^	**40.02**	<1.0 × 10^−4^	**30.16**	<1.0 × 10^−4^
*NOD2*	Nucleotide binding oligomerization domain containing 2	7.6	919.4	96.7	43.9	**116.73**	<1.0 × 10^−4^	**13.67**	<1.0 × 10^−4^	**5.61**	<1.0 × 10^−4^
*PTX3*	Pentraxin 3	184.4	18,888.7	4615.3	479.6	**113.19**	<1.0 × 10^−4^	**27.47**	<1.0 × 10^−4^	**2.60**	<1.0 × 10^−4^
*SAA1*	Serum amyloid A1	66.6	2188.7	3520.0	227.6	**94.66**	<1.0 × 10^−4^	**183.26**	<1.0 × 10^−4^	**6.46**	<1.0 × 10^−4^
*CD300E*	CD300e molecule	3.6	316.9	32.7	71.6	**72.79**	<1.0 × 10^−4^	**7.91**	<1.0 × 10^−4^	**17.15**	<1.0 × 10^−4^
*IL36B*	Interleukin 36 beta	11.3	466.3	80.1	39.1	**67.27**	<1.0 × 10^−4^	**9.65**	<1.0 × 10^−4^	**3.60**	<1.0 × 10^−4^
*TNFRSF1B*	TNF receptor superfamily member 1B	40.0	2370.7	525.8	118.9	**62.58**	<1.0 × 10^−4^	**14.66**	<1.0 × 10^−4^	**3.02**	<1.0 × 10^−4^
*TNFAIP6*	TNF alpha induced protein 6	1176.4	42,950.3	5512.4	4561.2	**36.87**	<1.0 × 10^−4^	**4.59**	<1.0 × 10^−4^	**3.59**	<1.0 × 10^−4^
*TMEM132A*	Transmembrane protein 132A	10.3	328.1	165.0	32.6	**33.90**	<1.0 × 10^−4^	**16.64**	<1.0 × 10^−4^	**3.18**	<1.0 × 10^−4^
*ICAM1*	Intercellular adhesion molecule 1	1415.2	42,657.2	4388.3	8524.5	**31.66**	<1.0 × 10^−4^	**3.15**	<1.0 × 10^−4^	**5.54**	<1.0 × 10^−4^
*C3AR1*	Complement C3a receptor 1	2.2	66.2	11.4	11.2	**28.15**	<1.0 × 10^−4^	**6.36**	1.5 × 10^−4^	**5.32**	4.9 × 10^−4^
*CLEC2B*	C-type lectin domain family 2 member B	5.3	145.0	48.5	20.6	**27.53**	<1.0 × 10^−4^	**9.35**	<1.0 × 10^−4^	**3.85**	<1.0 × 10^−4^
*COX-2/PTGS2*	Cyclooxygenase-2/Prostaglandin-endoperoxide synthase 2	1310.7	37,281.5	4678.6	5349.2	**26.96**	<1.0 × 10^−4^	**3.28**	<1.0 × 10^−4^	**3.57**	<1.0 × 10^−4^
*TLR2*	Toll like receptor 2	134.9	3348.9	782.0	371.4	**22.64**	<1.0 × 10^−4^	**5.02**	<1.0 × 10^−4^	**2.54**	<1.0 × 10^−4^
*CCL7*	C-C motif chemokine ligand 7	2.1	36.7	20.6	24.4	**20.66**	<1.0 × 10^−4^	**12.14**	<1.0 × 10^−4^	**10.56**	<1.0 × 10^−4^
*CCL2*	C-C motif chemokine ligand 2	150.4	2475.0	815.0	430.6	**19.42**	<1.0 × 10^−4^	**5.85**	<1.0 × 10^−4^	**2.61**	<1.0 × 10^−4^
*IRF4*	Interferon regulatory factor 4	23.5	400.1	94.9	114.2	**18.20**	<1.0 × 10^−4^	**4.62**	<1.0 × 10^−4^	**4.69**	<1.0 × 10^−4^
*CD274*	CD274 molecule	61.8	1048.8	350.1	3845.7	**17.56**	<1.0 × 10^−4^	**6.18**	<1.0 × 10^−4^	**60.08**	<1.0 × 10^−4^
*RBM47*	RNA binding motif protein 47	8.8	122.3	30.6	22.8	**14.96**	<1.0 × 10^−4^	**3.38**	<1.0 × 10^−4^	**2.67**	0.040
*CD38*	CD38 molecule	9.8	133.8	74.3	211.4	**14.81**	<1.0 × 10^−4^	**7.67**	<1.0 × 10^−4^	**20.76**	<1.0 × 10^−4^
*BDKRB1*	Bradykinin receptor B1	29.0	401.5	129.6	105.0	**13.95**	<1.0 × 10^−4^	**4.88**	<1.0 × 10^−4^	**3.19**	<1.0 × 10^−4^
*GCH1*	GTP cyclohydrolase 1	591.7	7968.7	2212.7	3584.2	**13.38**	<1.0 × 10^−4^	**3.90**	<1.0 × 10^−4^	**5.63**	<1.0 × 10^−4^
*LRRC38*	Leucine rich repeat containing 38	11.2	132.1	44.4	35.8	**11.59**	<1.0 × 10^−4^	**3.79**	<1.0 × 10^−4^	**2.98**	<1.0 × 10^−4^
*KIAA1217*	KIAA1217	15.3	157.8	55.1	109.1	**10.61**	<1.0 × 10^−4^	**3.80**	<1.0 × 10^−4^	**6.39**	<1.0 × 10^−4^
*SSTR2*	Somatostatin receptor 2	90.0	971.2	1549.7	340.1	**10.56**	<1.0 × 10^−4^	**16.11**	<1.0 × 10^−4^	**3.36**	<1.0 × 10^−4^
*DUSP5*	Dual specificity phosphatase 5	77.3	746.8	302.4	236.1	**10.54**	<1.0 × 10^−4^	**4.02**	<1.0 × 10^−4^	**2.90**	<1.0 × 10^−4^
*TYMP*	Thymidine phosphorylase	311.3	3020.1	1275.1	9324.0	**10.15**	<1.0 × 10^−4^	**4.24**	<1.0 × 10^−4^	**28.71**	<1.0 × 10^−4^
*GPR158*	G protein-coupled receptor 158	6.9	38.0	22.0	21.5	**9.98**	<1.0 × 10^−4^	**6.77**	0.0018	**5.55**	7.6 × 10^−4^
*PRLR*	Prolactin receptor	8.3	78.8	29.7	33.0	**9.93**	<1.0 × 10^−4^	**3.05**	0.0034	**3.92**	<1.0 × 10^−4^
*GSAP*	Gamma-secretase activating protein	122.2	1109.8	378.0	509.3	**9.18**	<1.0 × 10^−4^	**3.26**	<1.0 × 10^−4^	**3.74**	< 1.0 × 10^−4^
*GPR39*	G protein-coupled receptor 39	15.4	110.6	39.1	41.4	**9.17**	<1.0 × 10^−4^	**3.24**	1.7 × 10^−4^	**2.71**	<1.0 × 10^−4^
*LYPD1*	LY6/PLAUR domain containing 1	10.5	71.5	28.7	27.7	**8.44**	<1.0 × 10^−4^	**3.31**	5.6 × 10^−4^	**2.62**	0.0023
*ODF3B*	Outer dense fiber of sperm tails 3B	34.6	261.0	106.0	773.8	**7.98**	<1.0 × 10^−4^	**3.28**	<1.0 × 10^−4^	**21.57**	<1.0 × 10^−4^
*SLC15A3*	Solute carrier family 15 member 3	16.3	119.4	54.7	607.4	**7.63**	<1.0 × 10^−4^	**3.45**	<1.0 × 10^−4^	**35.59**	<1.0 × 10^−4^
*HAL*	Histidine ammonia-lyase	6.2	44.1	28.7	47.4	**7.57**	<1.0 × 10^−4^	**4.71**	<1.0 × 10^−4^	**6.97**	<1.0 × 10^−4^
*DOCK4*	Dedicator of cytokinesis 4	44.0	306.8	144.9	139.2	**6.94**	<1.0 × 10^−4^	**3.21**	<1.0 × 10^−4^	**2.91**	<1.0 × 10^−4^
*RAB27B*	RAB27B, member RAS oncogene family	16.5	77.2	60.5	84.5	**5.98**	<1.0 × 10^−4^	**3.85**	<1.0 × 10^−4^	**5.62**	<1.0 × 10^−4^
*CH25H*	Cholesterol 25-hydroxylase	7.4	36.5	25.8	41.8	**4.41**	<1.0 × 10^−4^	**3.27**	0.022	**6.32**	<1.0 × 10^−4^
*USP43*	Ubiquitin specific peptidase 43	4.4	12.8	13.6	16.1	**3.94**	0.020	**3.41**	0.013	**4.50**	0.0091
*AC104966.1*	Ceruloplasmin (ferroxidase) (CP) pseudogene	16.5	47.6	57.3	53.7	**3.39**	<1.0 × 10^−4^	**3.79**	<1.0 × 10^−4^	**3.36**	<1.0 × 10^−4^
*KLK10*	Kallikrein related peptidase 10	14.0	37.1	33.0	43.1	**3.11**	0.022	**3.29**	0.0067	**2.65**	0.0028

Red = upregulated genes.

**Table 6:** Genes downregulated by all studied proinflammatory cytokines with FC < −2.5. Shown are mean normalized expression levels in control (Co), in C(IL1), C(IL17) and C(IFNγ) phenotypes, fold changes (FCs) for all comparisons vs. control and false discovery rate (FDR)-adjusted *p* values for them.

Gene	Name	Function	Mean exp. (Co)	Mean exp. (IL1)	Mean exp. (IL17)	Mean exp. (IFNγ)	FC (IL1 vs. Co)	adj. p (IL1 vs. Co)	FC (IL17 vs. Co)	adj. p (IL17 vs. Co)	FC (IFNγ vs. Co)	adj. p (IFNγ vs. Co)
*SCN2B*	Sodium voltage-gated channel beta subunit 2	Sodium ion transport	170.8	8.7	65.9	63.7	**−22.19**	<1.0 × 10^−4^	**−2.59**	<1.0 × 10^−4^	**−2.90**	<1.0 × 10^−4^
*TNNT3*	Troponin T3, fast skeletal type	Skeletal muscle constituent	97.8	6.4	17.9	31.6	**−17.51**	<1.0 × 10^−4^	**−5.28**	<1.0 × 10^−4^	**−3.26**	<1.0 × 10^−4^
*MRAP2*	Melanocortin 2 receptor accessory protein 2	Metabolism?	1348.7	91.1	376.5	572.0	**−15.12**	<1.0 × 10^−4^	**−3.62**	<1.0 × 10^−4^	**−2.85**	<1.0 × 10^−4^
*WFDC1*	WAP four-disulfide core domain 1	Negative regulation of cell growth	60.1	6.1	34.9	18.0	**−12.06**	<1.0 × 10^−4^	**−2.68**	0.0019	**−4.06**	<1.0 × 10^−4^
*RANBP3L*	RAN binding protein 3 like	Nuclear export	654.8	74.6	284.8	280.0	**−9.40**	<1.0 × 10^−4^	**−2.54**	<1.0 × 10^−4^	**−2.60**	<1.0 × 10^−4^
*ASPN*	Asporin	Skeletal system development, negative regulation of TGFβ signaling	2094.0	206.3	505.2	837.5	**−8.28**	<1.0 × 10^−4^	**−3.92**	<1.0 × 10^−4^	**−2.77**	<1.0 × 10^−4^
*FGFR4*	Fibroblast growth factor receptor 4	Cell proliferation and migration	10.3	2.3	3.1	5.1	**−5.59**	5.2 × 10^−4^	**−3.12**	0.036	**−3.31**	0.045
*PTGER3*	Prostaglandin E receptor 3	Inflammation, cell death	494.1	173.6	162.3	188.8	**−2.69**	<1.0 × 10^−4^	**−3.03**	<1.0 × 10^−4^	**−2.82**	<1.0 × 10^−4^

blue = downregulated genes.

## Data Availability

Complete gene expression data for all samples are available from the corresponding author upon reasonable request.

## Supplementary Materials

The following are available online at https://www.mdpi.com/article/10.3390/ijms22179463/s1.
