# Supplementary material for: Chondrocytes from Osteoarthritis Patients Adopt Distinct Phenotypes in Response to Central TH1/TH2/TH17 Cytokines
Source: Int J Mol Sci. 2021 Aug 31;22(17):9463. doi: 10.3390/ijms22179463 (PMC8431052; doi:10.3390/ijms22179463)
Supplement: Supplementary file 1 [file ijms-22-09463-s001.zip › ijms-1302126 SI.pdf]

Table S1: Twenty most strongly upregulated genes in interleukin 4 -treated OA chondrocytes (IL4) relative to control (Co).

**Gene**	**Name**	**Function**	**Mean (Co)**	**Mean (IL4)**	**Fold Change**	**adj. p**
KCNK3	Potassium two pore domain channel subfamily K member 3	Potassium ion transport	4.2	41.0	**12.97**	$<1.0 \times 10^{-4}$
FGL2	Fibrinogen like 2	Negative regulation of antigen presentation?	26.8	361.2	**10.00**	$<1.0 \times 10^{-4}$
SOCS1	Suppressor of cytokine signaling 1	Regulation of inflammation	4.4	37.0	**8.99**	$<1.0 \times 10^{-4}$
MAOA	Monoamine oxidase A	Dopamine catabolism	17.8	157.1	**8.56**	$<1.0 \times 10^{-4}$
CSN1S1	Casein alpha s1	Steroid signaling?	6.8	20.8	**6.16**	2.5×10^{-4}
LRRC32	Leucine rich repeat containing 32	TGFβ signaling	6.2	36.5	**5.43**	5.30×10^{-5}
FAM19A1	Family with sequence similarity 19 member A1, C-C motif chemokine like	Neuroblast differentiation?	4.0	17.5	**5.04**	0.0059
OTOGL	Otogelin like	?	3.8	17.5	**4.86**	3.2×10^{-4}
SUCNR1	Succinate receptor 1	Metabolism and inflammation	3.6	15.1	**4.65**	0.0018
ANPEP	Alanyl aminopeptidase, membrane	Cell adhesion and inflammation	88.3	408.8	**4.41**	$<1.0 \times 10^{-4}$
CADPS	Calcium dependent secretion activator	Exocytosis	4.1	11.3	**4.16**	0.047
SH2D1B	SH2 domain containing 1B	Regulation of inflammation	7.3	27.7	**4.12**	2.90×10^{-5}
CISH	Cytokine inducible SH2 containing protein	Regulation of cell growth and inflammation	16.0	64.6	**4.08**	2.3×10^{-4}
SELP	Selectin P	Cell adhesion and inflammation	202.7	849.8	**4.00**	$<1.0 \times 10^{-4}$
SAMD3	Sterile alpha motif domain containing 3	?	4.3	17.3	**3.95**	0.0066
CAPN8	Calpain 8	?	3.0	10.9	**3.85**	0.011
LPL	Lipoprotein lipase	Lipid metabolism and inflammation	15.1	44.1	**3.65**	1.0×10^{-5}
CYTIP	Cytohesin 1 interacting protein	Cell adhesion	8.9	33.8	**3.61**	4.80×10^{-5}
RASL11B	RAS like family 11 member B	TGFβ signaling	4.6	17.9	**3.44**	0.018
MT-TW	Mitochondrially encoded trna tryptophan	?	24.7	60.6	**3.38**	2.3×10^{-4}

Table S2: Genes downregulated with FC < −1.5 in interleukin 4 -treated OA chondrocytes (IL4) relative to control (Co)

Gene	Name	Function	Mean (Co)	Mean (IL4)	Fold Change	adj. p
OSR1	Odd-skipped related transciption factor 1	Regulation of cell proliferation	50.2	28.6	**−1.95**	0.016
ANGPTL7	Angiopoietin like 7	Regulation of angiogenesis	572.7	403.0	**−1.72**	0.019
SCG5	Secretogranin V	Regulation of protein aggregation	164.7	109.6	**−1.59**	0.035
OLFML2B	Olfactomedin like 2B	?	84.9	50.7	**−1.56**	0.025
NEK2	NIMA related kinase 2	Regulation of cell proliferation	420.4	295.1	**−1.55**	2.1×10^{-4}
HIST1H4L	Histone cluster 1 H4 family member l	?	178.3	136.3	**−1.54**	0.026
NDC80	NDC80, kinetochore complex component	Cell division	821.8	578.4	**−1.53**	5.0×10^{-6}
RORC	RAR related orphan receptor C	Regulation of cell differentiation and inflammation?	148.9	105.2	**−1.52**	0.027
AURKB	Aurora kinase B	Regulation of cell proliferation	164.0	114.5	**−1.52**	0.0044
HMMR-AS1	HMMR antisense RNA 1	Regulation of cell motility?	248.4	173.2	**−1.50**	5.4×10^{-4}

Table S3: GO terms enriched among the genes significantly affected by IL-4. The list of genes with FC > 1.5 in either direction and with FDR-corrected p-value < 0.05 was analyzed with DAVID, and the resulting list of GO terms was reduced with REVIGO.

**Term**	**adj. p**
Cell division	2.45×10^{-11}
Chromosome segregation	9.74×10^{-8}
Microtubule-based movement	6.36×10^{-6}
CENP-A containing nucleosome assembly	0.00035
Protein localization to kinetochore	0.00099
Regulation of gene silencing	0.0014
Metaphase plate congression	0.0018
Protein heterotetramerization	0.0027
G2/M transition of mitotic cell cycle	0.0029
Telomere organization	0.0030
DNA replication-dependent nucleosome assembly	0.0064
Positive regulation of cytokinesis	0.012
Positive regulation of gene expression, epigenetic	0.017
Blood coagulation	0.023
DNA replication	0.026
Anaphase-promoting complex-dependent catabolic process	0.049
Cell proliferation	0.049

Table S4: Genes markedly (FC > 2.5) upregulated in degraded OA cartilage compared to preserved in Almeida et al. [35] and significantly affected by proinflammatory cytokines in our study.

Gene	Name	FC (Almeida)	FDR (Almeida)	FC (IL1β)	adj. p (IL1β)	FC (IL17)	adj. p (IL17)	FC (IFNγ)	adj. p (IFNγ)
IL11	Interleukin 11	**22.8**	1.53×10^{-20}	**7.63**	<0.0001	**3.31**	0.00043	-	-
IGFBP1	Insulin like growth factor binding protein 1	**7.59**	1.32×10^{-9}	**3.65**	<0.0001	**3.06**	<0.0001	**1.47**	<0.0001
TNP1	Transition protein 1	**6.6**	2.60×10^{-5}	**15.70**	<0.0001	**5.23**	0.00019	-	-
ISM2	Isthmin 2	**5.24**	4.71×10^{-18}	-	-	-	-	**3.90**	<0.0001
RIPK4	Receptor interacting serine/threonine kinase 4	**5.22**	5.45×10^{-13}	**5.13**	<0.0001	**2.12**	0.00076	-	-
KPNA7	Karyopherin subunit alpha 7	**5.1**	7.56×10^{-6}	**4.99**	<0.0001	-	-	**2.42**	0.0052
LIF	LIF, interleukin 6 family cytokine	**4.91**	7.79×10^{-10}	**30.92**	<0.0001	**12.51**	<0.0001	**1.59**	<0.0001
NGF	Nerve growth factor	**4.91**	2.53×10^{-14}	**4.86**	<0.0001	-	-	**1.37**	0.00027
TFPI2	Tissue factor pathway inhibitor 2	**4.08**	9.92×10^{-9}	**26.93**	<0.0001	**2.41**	<0.0001	**1.27**	0.0086
LAMC2	Laminin subunit gamma 2	**3.75**	2.05×10^{-6}	**9.63**	<0.0001	-	-	-	-
CLIC3	Chloride intracellular channel 3	**3.65**	1.81×10^{-11}	**−3.23**	0.00029	-	-	**2.15**	<0.0001
TNFAIP6	TNF alpha induced protein 6	**3.58**	2.48×10^{-8}	**36.87**	<0.0001	**4.59**	<0.0001	**3.59**	<0.0001
ERFE	Erythroferrone	**3.44**	8.82×10^{-12}	**−3.36**	<0.0001	**1.41**	0.004	**−1.62**	<0.0001
PAPPA	Pappalysin 1	**3.43**	1.14×10^{-10}	**6.46**	<0.0001	**1.39**	0.00015	**1.57**	<0.0001
DNER	Delta/notch like EGF repeat containing	**3.37**	4.24×10^{-11}	**4.49**	<0.0001	**2.66**	<0.0001	-	-
CCL20	C-C motif chemokine ligand 20	**3.35**	5.10×10^{-5}	**1128.35**	<0.0001	**73.49**	<0.0001	-	-
HMGA2	High mobility group AT-hook 2	**3.22**	3.85×10^{-7}	**8.04**	<0.0001	**5.34**	<0.0001	**1.51**	0.0018
BMPR1B	Bone morphogenetic protein receptor type 1B	**3.2**	1.28×10^{-7}	**6.94**	<0.0001	**2.09**	0.0049	-	-
LAMB3	Laminin subunit beta 3	**3.11**	7.66×10^{-13}	**20.72**	<0.0001	**3.42**	<0.0001	-	-
PTGES	Prostaglandin E synthase	**3.06**	3.61×10^{-12}	**5.72**	<0.0001	**4.27**	<0.0001	**2.04**	<0.0001
KRT16	Keratin 16	**3.03**	1.22×10^{-5}	**36.58**	<0.0001	-	-	-	-
TNFRSF11B	TNF receptor superfamily member 11b	**3.01**	7.12×10^{-12}	**2.74**	<0.0001	**3.12**	<0.0001	**−1.25**	0.00021
SOX11	SRY-box 11	**2.99**	1.62×10^{-8}	**3.64**	<0.0001	-	-	**−1.52**	0.017
DNAJC12	Dnaj heat shock protein family (Hsp40) member C12	**2.97**	5.10×10^{-5}	-	-	**3.33**	0.044	-	-
KRT17	Keratin 17	**2.96**	4.08×10^{-5}	**18.39**	<0.0001	-	-	**5.21**	<0.0001
TRIM36	Tripartite motif containing 36	**2.85**	1.08×10^{-8}	**7.06**	<0.0001	-	-	-	-
USP43	Ubiquitin specific peptidase 43	**2.77**	0.00014	**3.94**	0.02	**3.41**	0.013	**4.50**	0.0091
GPR158	G protein-coupled receptor 158	**2.73**	0.0036	**9.98**	2.50×10^{-5}	**6.77**	0.0018	**5.55**	0.00076
SHISA9	Shisa family member 9	**2.73**	4.47×10^{-5}	-	-	-	-	**−2.72**	2.90×10^{-5}
NTF3	Neurotrophin 3	**2.72**	6.59×10^{-6}	**2.98**	0.00053	-	-	-	-
FAM167A	Family with sequence similarity 167 member A	**2.67**	1.91×10^{-7}	**3.54**	<0.0001	**2.09**	0.035	-	-
TREM1	Triggering receptor expressed on myeloid cells 1	**2.66**	7.77×10^{-5}	**12.14**	<0.0001	**2.47**	<0.0001	**−1.68**	6.70×10^{-5}
IGFBP3	Insulin like growth factor binding protein 3	**2.65**	1.12×10^{-7}	**2.87**	<0.0001	**1.89**	<0.0001	**−1.16**	0.001
SLC27A2	Solute carrier family 27 member 2	**2.64**	7.84×10^{-5}	**−2.59**	0.024	-	-	**−2.52**	0.0073
ANK3	Ankyrin 3	**2.62**	6.02×10^{-10}	**−6.74**	<0.0001	**−3.02**	<0.0001	**−1.91**	<0.0001

DAW1	Dynein assembly factor with WD repeats 1	**2.57**	2.55×10^{-5}	**2.96**	8.00×10^{-6}	**2.44**	<0.0001	**−1.76**	0.022
ANXA8	Annexin A8	**2.56**	4.84×10^{-6}	**31.85**	4.20×10^{-5}	**-**	-	**-**	-
KLHL30-AS1	Kelch like family member 30	**2.5**	0.0037	**-**	-	**4.31**	0.0067	**-**	-

Red = upregulated genes
Blue = downregulated genes

Table S5: Genes markedly (FC < −2.5) downregulated in degraded OA cartilage compared to preserved in Almeida et al. [35] and significantly affected by proinflammatory cytokines in our study.

Gene	Name	FC (Almeida)	FDR (Almeida)	FC (IL1β)	adj. p (IL1β)	FC (IL17)	adj. p (IL17)	FC (IFNγ)	adj. p (IFNγ)
RCAN2	Regulator of calcineurin 2	**−4.08**	9.19×10^{-12}	**−16.58**	<0.0001	**−4.74**	<0.0001	**−1.46**	0.00015
VIT	Vitrin	**−3.91**	1.14×10^{-8}	**−2.96**	<0.0001	**−1.44**	0.0008	**−1.95**	<0.0001
C3	Complement C3	**−3.83**	4.00×10^{-7}	**67.78**	<0.0001	**33.70**	<0.0001	**-**	-
SHISA3	Shisa family member 3	**−3.67**	3.14×10^{-8}	**−10.46**	<0.0001	**−2.11**	<0.0001	**−1.35**	5.1×10^{-5}
RSPO3	R-spondin 3	**−3.62**	1.48×10^{-6}	**47.37**	<0.0001	**4.42**	<0.0001	**2.08**	<0.0001
PTGER3	Prostaglandin E receptor 3	**−3.08**	4.91×10^{-8}	**−2.69**	<0.0001	**−3.03**	<0.0001	**−2.82**	<0.0001
MATN4	Matrilin 4	**−3.04**	0.0028	**−2.59**	0.022	**-**	-	**-**	-
GDF10	Growth differentiation factor 10	**−2.86**	1.05×10^{-8}	**−20.57**	<0.0001	**−2.93**	<0.0001	**−1.70**	<0.0001
GDF7	Growth differentiation factor 7	**−2.81**	1.91×10^{-6}	**−2.60**	0.0001	**-**	-	**-**	-
NCMAP	Non-compact myelin associated protein	**−2.80**	0.0012	**-**	-	**4.08**	<0.0001	**-**	-
CRISPLD1	Cysteine rich secretory protein LCCL domain containing 1	**−2.76**	9.29×10^{-6}	**−5.00**	<0.0001	**−3.39**	<0.0001	**-**	-
KIF1A	Kinesin family member 1A	**−2.70**	8.64×10^{-8}	**4.59**	0.00042	**-**	-	**-**	-
DACT1	Dishevelled binding antagonist of beta catenin 1	**−2.68**	2.85×10^{-7}	**−4.13**	<0.0001	**-**	-	**1.26**	0.0079
GREM1	Gremlin 1, DAN family BMP antagonist	**−2.65**	2.57×10^{-5}	**−8.76**	<0.0001	**−3.41**	<0.0001	**-**	-
ZFHX4-AS1	ZFHX4 antisense RNA 1	**−2.65**	0.021	**−6.04**	0.0001	**−2.77**	0.012	**-**	-
KIT	KIT proto-oncogene receptor tyrosine kinase	**−2.58**	0.00052	**3.50**	0.0072	**-**	-	**-**	-
ERICH3	Glutamate rich 3	**−2.56**	0.023	**−5.62**	<0.0001	**-**	-	**−2.64**	0.0012
PRLR	Prolactin receptor	**−2.55**	0.019	**9.93**	<0.0001	**3.05**	0.0034	**3.92**	8.3×10^{-5}
STEAP4	STEAP4 metalloreductase	**−2.53**	4.95×10^{-9}	**−3.52**	<0.0001	**-**	-	**−1.79**	<0.0001

Red = upregulated genes
Blue = downregulated genes

Table S6: Genes markedly differentially expressed in degraded and preserved OA cartilage (with FC > 1.5 in either direction) in Almeida et al. [35] and markedly affected (with FC > 1.5 in either direction) by IL-4 in our study.

Gene	Name	FC (Almeida)	adj. p (Almeida)	FC (our study)	adj. p (our study)
Genes upregulated in degraded cartilage (Almeida et al. [35]) and upregulated by IL-4 (our study)					
PTPRZ1	Protein tyrosine phosphatase, receptor type Z1	**1.94**	0.019	**2.62**	0.0074
TMEM200A	Transmembrane protein 200A	**1.87**	0.0014	**2.58**	<0.0001
POSTN	Periostin	**2.06**	0.032	**2.32**	<0.0001
TENM3	Teneurin transmembrane protein 3	**1.90**	4.08×10^{-5}	**2.08**	1.40×10^{-5}
TTC9	Tetratricopeptide repeat domain 9	**2.38**	2.30×10^{-7}	**2.07**	1.00×10^{-6}
DUSP5	Dual specificity phosphatase 5	**1.51**	0.031	**1.97**	0.0011
ANGPTL4	Angiopoietin like 4	**1.91**	8.50×10^{-6}	**1.67**	8.00×10^{-6}
LRRC8C	Leucine rich repeat containing 8 family member C	**2.36**	5.88×10^{-8}	**1.65**	0.00029
F5	Coagulation factor V	**1.68**	0.00084	**1.50**	0.04
COL7A1	Collagen type VII alpha 1 chain	**2.37**	1.96×10^{-8}	**1.50**	0.0019
Genes upregulated in degraded cartilage (Almeida et al. [35]) and downregulated by IL-4 (our study)					
HMMR	HMMR antisense RNA 1	**1.78**	0.047	**−1.50**	0.00054
Genes downregulated in degraded cartilage (Almeida et al. [35]) and upregulated by IL-4 (our study)					
FGL2	Fibrinogen like 2	**−1.71**	0.0093	**10.00**	<0.0001
CISH	Cytokine inducible SH2 containing protein	**−2.24**	4.71×10^{-18}	**4.08**	0.00023
GPM6A	Glycoprotein M6A	**−1.73**	0.015	**2.25**	0.044
COL14A1	Collagen type XIV alpha 1 chain	**−1.75**	0.033	**2.11**	<0.0001
CYSLTR1	Cysteinyl leukotriene receptor 1	**−1.68**	0.00085	**1.96**	0.00072
DKK1	Dickkopf WNT signaling pathway inhibitor 1	**−1.66**	0.018	**1.86**	0.028
CORO2B	Coronin 2B	**−1.82**	0.0001	**1.60**	0.014

Red = upregulated genes
Blue = downregulated genes

Table S7: Expression of central cytokine receptors in unstimulated chondrocytes. Mean expression values are given as DESeq2-normalized counts.

Gene	Mean (Co)
IL1R1	2448.7
IL4R	65.6
IL17RB	210.5
IL17RC	349.1
IL17RD	66.1
IL17RE	14.3
IFNGR1	1529.5
IFNGR2	1501.3
